# Real-world effectiveness and safety of goserelin 10.8-mg depot in Chinese patients with localized or locally advanced prostate cancer

**DOI:** 10.20892/j.issn.2095-3941.2023.0335

**Published:** 2024-02-05

**Authors:** Nanhui Chen, Zengjun Wang, Ming Chen, Qi Ma, Yi He, Yujie Wang, Xin Li, Mingxing Qiu, Lei Shi, Shaoxing Zhu, Qun Xie, Xiuheng Liu, Benkang Shi, Guowen Lin, Weizhong Yang, Yongbin Liao, Haibin Zhang, Shusheng Wang, Jiexian Li, Shaogang Wang, Lijun Dong, Hui Chen, Jiaju Lu, Yongyi Cheng, Xiaoping Zhang, Lulin Ma, Liqun Zhou, He Wang, Shen Li, Dingwei Ye

**Affiliations:** 1 Department of Urology, Meizhou People’s Hospital, Meizhou 514089, China; 2 Department of Urology, Jiangsu Province Hospital, Nanjing 210029, China; 3 Department of Urology, Zhongda Hospital Southeast University, Nanjing 210009, China; 4 Translational Research Laboratory for Urology, Ningbo First Hospital, Ningbo 315016, China; 5 Department of Urology, First Affiliated Hospital of Jiaxing, Jiaxing 314050, China; 6 Department of Urology, The First Affiliated Hospital of Xinjiang Medical University, Urumqi 830091, China; 7 Department of Urology, Baotou Cancer Hospital, Baotou 014016, China; 8 Department of Urology, The People’s Hospital of Sichuan Province, Chengdu 610032, China; 9 Department of Urology, Yantai Yuhuangding Hospital, Yantai 264008, China; 10 Department of Urology, Cancer Hospital of The University of Chinese Academy of Sciences, Hangzhou 310022, China; 11 Department of Urology, Zhuhai Hospital Affiliated with Jinan University, Zhuhai 519000, China; 12 Department of Urology, Tongji Hospital, Tongji Medical College, HUST, Wuhan 430032, China; 13 Department of Urology, Qilu Hospital of Shandong University, Jinan 250012, China; 14 Department of Urology, Fudan University Shanghai Cancer Center, Shanghai 200032, China; 15 Department of Urology, Huizhou Third People’s Hospital, Huizhou 516002, China; 16 Department of Urology, Jiangmen Central Hospital, Jiangmen 529030, China; 17 Department of Urology, Foshan First People’s Hospital, Foshan 528041, China; 18 Department of Urology, Guangzhou Province Traditional Chinese Medical Hospital, Guangzhou 510120, China; 19 Department of Urology, Guangzhou Panyu Central Hospital, Guangzhou 511489, China; 20 Department of Urology, Renmin Hospital of Wuhan University, Wuhan 430064, China; 21 Department of Urology, Hebei Petro China Central Hospital (China National Petroleum Corporation Central Hospital), Langfang 065099, China; 22 Department of Urology, Harbin Medical University Cancer Hospital, Harbin 150086, China; 23 Department of Urology, Shandong Provincial Hospital, Jinan 250014, China; 24 Department of Urology, Shaanxi Provincial People’s Hospital, Xi’an 710021, China; 25 Department of Urology, Union Hospital Tongji Medical College Huazhong University of Science and Technology, Wuhan 430032, China; 26 Department of Urology, Peking University Third Hospital, Beijing 100191, China; 27 Department of Urology, Peking University First Hospital, Beijing 100034, China; 28 Department of Urology, Tangdu Hospital, Xi’an 710024, China; 29 Department of Urology, Shijiazhuang City First Hospital, Shijiazhuang 050012, China

**Keywords:** Goserelin, hormone-sensitive, luteinizing hormone-releasing hormone, prostate cancer, China, real-world

## Abstract

**Objective:**

Real-word data on long-acting luteinizing hormone-releasing hormone (LHRH) agonists in Chinese patients with prostate cancer are limited. This study aimed to determine the real-world effectiveness and safety of the LHRH agonist, goserelin, particularly the long-acting 10.8-mg depot formulation, and the follow-up patterns among Chinese prostate cancer patients.

**Methods:**

This was a multicenter, prospective, observational study in hormone treatment-naïve patients with localized or locally advanced prostate cancer who were prescribed goserelin 10.8-mg depot every 12 weeks or 3.6-mg depot every 4 weeks with or without an anti-androgen. The patients had follow-up evaluations for 26 weeks. The primary outcome was the effectiveness of goserelin in reducing serum testosterone and prostate-specific antigen (PSA) levels. The secondary outcomes included testosterone and PSA levels, attainment of chemical castration (serum testosterone <50 ng/dL), and goserelin safety. The exploratory outcome was the monitoring pattern for serum testosterone and PSA. All analyses were descriptive.

**Results:**

Between September 2017 and December 2019, a total of 294 eligible patients received ≥ 1 dose of goserelin; 287 patients (97.6%) were treated with goserelin 10.8-mg depot. At week 24 ± 2, the changes from baseline [standard deviation (95% confidence interval)] in serum testosterone (*n* = 99) and PSA (*n* = 131) were −401.0 ng/dL [308.4 ng/dL (−462.5, −339.5 ng/dL)] and −35.4 ng/mL [104.4 ng/mL (−53.5, −17.4 ng/mL)], respectively. Of 112 evaluable patients, 100 (90.2%) achieved a serum testosterone level < 50 ng/dL. Treatment-emergent adverse events (TEAEs) and severe TEAEs occurred in 37.1% and 10.2% of patients, respectively. The mean testing frequency (standard deviation) was 1.6 (1.5) for testosterone and 2.2 (1.6) for PSA.

**Conclusions:**

Goserelin 10.8-mg depot effectively achieved and maintained castration and was well-tolerated in Chinese patients with localized and locally advanced prostate cancer.

## Introduction

Prostate cancer was the second most frequent and 5^th^ leading cause of death in all male cancers worldwide in 2020, with approximately 1.4 million new cases and 375,000 related deaths^1^. China has been faced with an increasing burden of prostate cancer, with an estimated 153,448 incident cases and 54,391 deaths in 2019^2^. Chinese patients with prostate cancer are older at the time of diagnosis, have higher pre-operative prostate specific antigen (PSA) levels, and are more likely to be diagnosed with advanced disease than their Western counterparts, suggesting that prostate cancer is more aggressive in Chinese patients^3,4^. This finding could possibly explain the lower 5-year survival rate in Chinese patients with prostate cancer compared to Western patient populations^5,6^.

Androgen deprivation therapy (ADT) has long been a mainstay of treatment for advanced prostate cancer. ADT is recommended as a first-line treatment for locally advanced or metastatic prostate cancer and as adjuvant therapy after radical prostatectomy (RP) for localized and locally advanced prostate cancer^7–10^. ADT consists of surgical castration (orchiectomy) and chemical castration with luteinizing hormone-releasing hormone (LHRH) agonists or antagonists^8^. Compared to surgical castration, chemical castration is associated with similar survival benefits but better quality-of-life outcomes and thus is more widely used^11–13^.

LHRH agonists are known to stimulate LHRH receptors on gonadotrophs that are located in the pituitary gland and secrete gonadotropins, such as luteinizing hormone (LH) and follicle-stimulating hormone^14^. LH stimulates Leydig cells in the testis to produce testosterone, which causes a surge in testosterone after initial LHRH agonist injection^14^. However, continuous administration of LHRH agonists downregulates LHRH receptors, resulting in decreased gonadotropin secretion and ultimately reduced testosterone production^14^. Long-acting LHRH agonists reduce injection frequency and thus provide better convenience for patients and healthcare professionals than short-acting LHRH agonists^15^. Despite these advantages, 3-month formulations of LHRH agonists are prescribed less frequently than 1-month formulations (38% *vs.* 62%) for patients with prostate cancer in China^16^.

Goserelin is an LHRH agonist commonly used to treat prostate cancer. The long-acting 10.8-mg depot (administered every 12 weeks) has been shown to have similar efficacy and safety to the 3.6-mg depot (administered every 4 weeks) according to several randomized controlled trials from The Netherlands, the US, and Japan^15,17–21^. However, long-acting goserelin 10.8-mg depot efficacy and safety data specifically involving Chinese patients are limited. The follow-up pattern of Chinese patients with prostate cancer undergoing ADT is also unknown.

Our study aimed to establish the real-world effectiveness and safety profiles of goserelin 10.8-mg depot in Chinese patients from 29 study centers with hormone treatment-naïve, localized, or locally advanced prostate cancer and to examine the real-world testing patterns of serum testosterone and prostate-specific antigen (PSA) while under goserelin treatment.

## Materials and methods

### Study design and participants

This was a multicenter, prospective, observational study conducted at 29 study centers in China. Male patients who met the following criteria were eligible for study enrollment: a) at least 18 years of age; b) locally advanced prostate cancer but have not undergone an RP or received a diagnosis of localized or locally advanced prostate cancer and underwent an RP; c) prescribed goserelin 10.8-mg depot every 12 weeks or 3.6-mg depot every 4 weeks as monotherapy or in combination with an anti-androgen; and d) have a life expectancy > 26 weeks. Patients with the following criteria were not eligible for study enrollment: a) planned to receive radiation therapy as radical treatment (adjuvant radiation therapy combined with goserelin after RP was acceptable) or neoadjuvant hormone therapy after an RP; b) hypersensitivity to LHRH, its analogues, or any components of goserelin depot; and c) received previous or concurrent hormonal therapy, excluding traditional anti-androgen therapy 2 weeks before goserelin treatment.

The study was approved (approval No. 2016S000237) by the Ethics Committee of Shijiazhuang City First Hospital (Shijiazhuang, China). The study was performed in accordance with the Declaration of Helsinki and Good Clinical Practice. All patients provided informed consent prior to study enrolment.

### Procedures

All diagnosis and treatment decisions were made at the discretion of the investigators. The planned follow-up period was 26 weeks in length and the schedule was discussed and agreed upon between physicians and patients. Serum testosterone and PSA levels were measured at baseline and each follow-up visit, as recommended by the physicians. Adverse events (AEs) were coded according to the Medical Dictionary for Regulatory Activities (version 24.0) and graded using the Common Terminology Criteria for Adverse Events (version 5.0).

### Outcomes

The primary outcomes were changes from baseline in serum testosterone and PSA levels at each visit during the follow-up period. The secondary outcomes were safety, mean serum testosterone and PSA levels, and the number and proportion of patients who achieved chemical castration (serum testosterone < 50 ng/dL) at each follow-up visit. The frequencies of serum testosterone and PSA testing during follow-up were defined as exploratory outcomes.

To investigate the clinical efficacy of goserelin in distinct patient populations with highly heterogeneous clinical features, prespecified subgroup efficacy analyses were conducted based on patient disease status (localized or locally advanced) and RP status (with or without RP). Furthermore, to explore the potential impact of concurrent anti-androgen therapy on the efficacy of goserelin, another prespecified subgroup efficacy analysis was performed based on treatment strategy (with or without the anti-androgen, bicalutamide).

### Statistical analysis

This study had no formal sample size calculation because the study design was observational without pre-specified hypotheses. Three hundred and twenty patients were planned to be enrolled based on site capacity within a 1-year recruitment period. Prespecified efficacy analyses were conducted for 6 timepoints (weeks 4 ± 2, 8 ± 2, 12 ± 2, 16 ± 2, 20 ± 2, and 24 ± 2) in the full analysis set (FAS), which consisted of all patients who met the eligibility criteria and received ≥ 1 dose of goserelin. The 95% confidence intervals (CIs) for mean changes from baseline in serum testosterone and PSA levels at weeks 12 ± 2 and 24 ± 2 were calculated using the *t*-distribution. A *post-hoc* analysis of serum testosterone levels was performed in the testosterone subset, which consisted of all patients who completed the 3 serum tests for testosterone at baseline, week 12 ± 2, and week 24 ± 2. A similar *post-hoc* analysis was performed for PSA levels in the PSA subset, which consisted of patients who underwent the 3 PSA tests at baseline, week 12 ± 2, and week 24 ± 2. Another *post-hoc* analysis of the castration rate in patients who received goserelin 10.8-mg depot was performed for 4 timepoints (between the first and second injection, after the second injection, and 28–84 days after the first and second injections). The safety analysis was conducted in the FAS. All analyses were descriptive and performed using SAS Enterprise Guide^®^ (version 7.1).

## Results

### Study population

**Figure 1:**
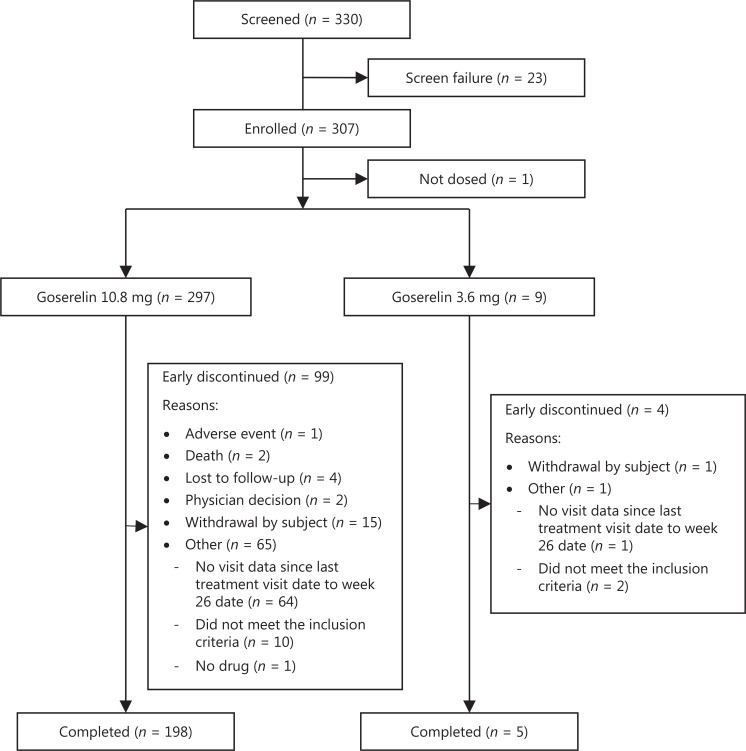
Flow diagram.

The first patient was enrolled in September 2017 and the last study visit was completed in December 2019. Of 330 patients screened, 307 were enrolled and 294 were included in the FAS (**Figure 1**). Patient demographics and baseline characteristics are summarized in **Table 1**. The testosterone (*n* = 80) and PSA subsets (*n* = 107) had demographics and baseline characteristics similar to the FAS (**Table 1**). One hundred thirty-five (45.9%) and 159 patients (54.1%) in the FAS were diagnosed with localized and locally advanced prostate cancer, respectively. A total of 182 (61.9%) patients underwent an RP and all received goserelin 10.8-mg depot as adjuvant therapy. One hundred twelve patients (38.1%) did not undergo an RP and received goserelin 10.8-mg (*n* = 105) or 3.6-mg depot (*n* = 7) as first-line treatment. Of the 182 patients who underwent an RP, 132 (72.5%) received bicalutamide in addition to goserelin, and 109 of the 112 patients (97.3%) who did not undergo an RP received bicalutamide add-on.

**Table 1:** Baseline demographics and characteristics in the full analysis set, the testosterone subset, and the PSA subset

Characteristics	FAS (*n* = 294)	Testosterone subset^†^ (*n* = 80)	PSA subset^†^ (*n* = 107)
Mean age ± SD, years	70.2 ± 7.7	68.9 ± 6.9	68.9 ± 6.6
ECOG score, *n* (%)			
0	152 (51.7)	47 (58.8)	58 (54.2)
1	131 (44.6)	32 (40.0)	46 (43.0)
2–3	11 (3.7)	1 (1.3)	3 (2.8)
Mean serum PSA ± SD, ng/mL	36.7 ± 88.8	–	39.5 ± 116.4
Mean serum testosterone ± SD, ng/dL	574.5 ± 2,053.5	392.2 ± 169.8	–
Disease status, *n* (%)			
Localized	135 (45.9)	35 (43.8)	49 (45.8)
Locally advanced	159 (54.1)	45 (56.3)	58 (54.2)
RP status, *n* (%)			
RP	182 (61.9)	57 (71.3)	80 (74.8)
No RP	112 (38.1)	23 (28.8)	27 (25.2)
T staging, *n* (%)			
T1–2	108 (36.7)	31 (38.8)	39 (36.4)
T3–4	161 (54.8)	45 (56.3)	60 (56.1)
Missing	25 (8.5)	4 (5.0)	8 (7.5)
N staging, *n* (%)			
N0	221 (75.2)	60 (75.0)	79 (73.8)
N1	35 (11.9)	12 (15.0)	15 (14.0)
Nx	18 (6.1)	7 (8.8)	7 (6.5)
Missing	20 (6.8)	1 (1.3)	6 (5.6)
Gleason score, *n* (%)			
6	19 (6.5)	4 (5.0)	6 (5.6)
7	110 (37.4)	30 (37.5)	43 (40.2)
8	77 (26.2)	18 (22.5)	25 (23.4)
≥ 9	76 (25.9)	23 (28.8)	27 (25.2)
Missing	3 (1.0)	1 (1.3)	1 (0.9)

^†^The testosterone and PSA subsets consisted of all patients who completed the three tests for serum testosterone and PSA, respectively, at baseline, week 12 ± 2, and week 24 ± 2. ECOG, Eastern Cooperative Oncology Group; PSA, prostate-specific antigen; RP, radical prostatectomy; SD, standard deviation.

### Effectiveness

#### Pre-specified analysis

**Figure 2:**
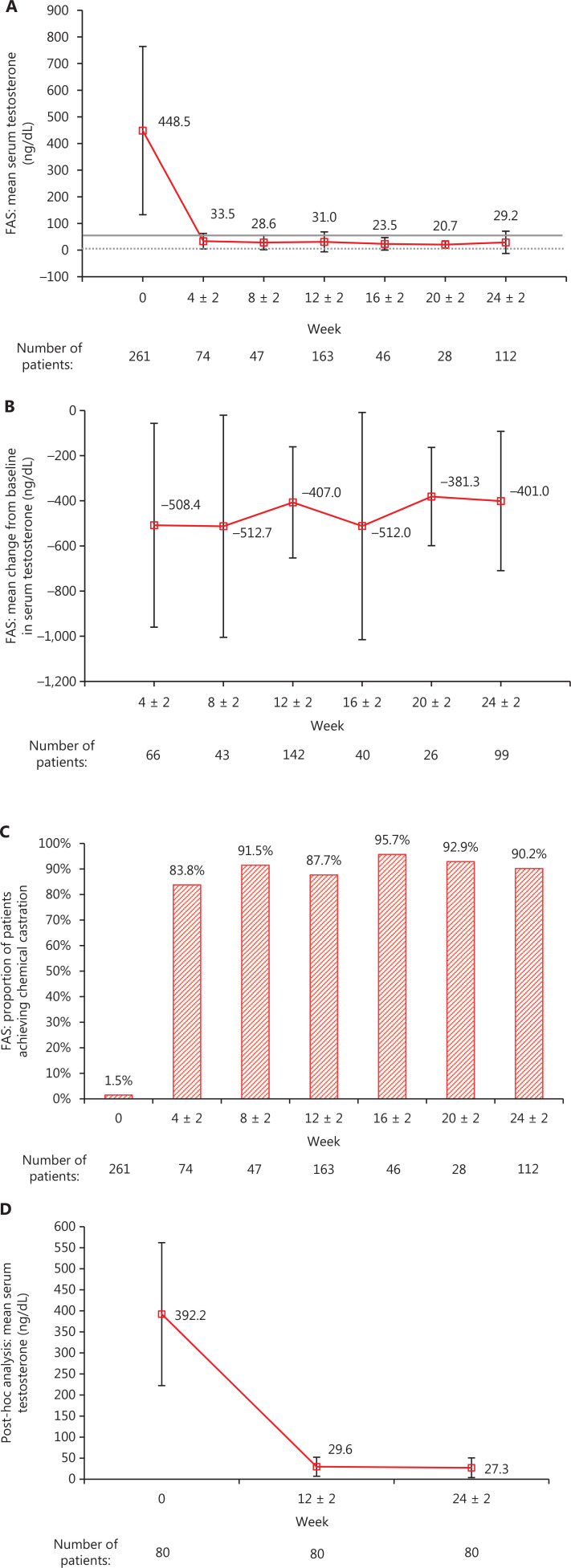
Serum testosterone: (A) mean (SD) serum testosterone level in the FAS; (B) mean (SD) change from baseline in serum testosterone level in the FAS; (C) proportion of patients with chemical castration (serum testosterone < 50 ng/dL) for the FAS; and (D) mean (SD) serum testosterone level in the testosterone subset. The gray dotted line in (A) represents zero, and the gray solid line in (A) represents the serum testosterone level of 50 ng/dL. FAS, full analysis set; SD, standard deviation.

The mean serum testosterone level [standard deviation (SD)] decreased from 448.5 ng/dL (315.9 ng/dL) at baseline (*n* = 261) to 33.5 ng/dL (29.2 ng/dL), 31.0 ng/dL (37.5 ng/dL), and 29.2 ng/dL (41.7 ng/dL) at weeks 4 ± 2 (*n* = 74), 12 ± 2 (*n* = 163), and 24 ± 2 (*n* = 112), respectively, in the FAS (**Figure 2A**). The mean change in serum testosterone level from baseline [SD (95 CI%)] was −407.0 ng/dL [246.1 ng/dL (−447.9, −366.2 ng/dL)] and −401.0 ng/dL [308.4 ng/dL (−462.5, −339.5 ng/dL)] at weeks 12 ± 2 (*n* = 142) and 24 ± 2 (*n* = 99), respectively (**Figure 2B**). At weeks 4 ± 2, 12 ± 2, and 24 ± 2, 83.8% (62/74), 87.7% (143/163), and 90.2% (100/112) of patients achieved chemical castration, respectively (**Figure 2C**).

**Figure 3:**
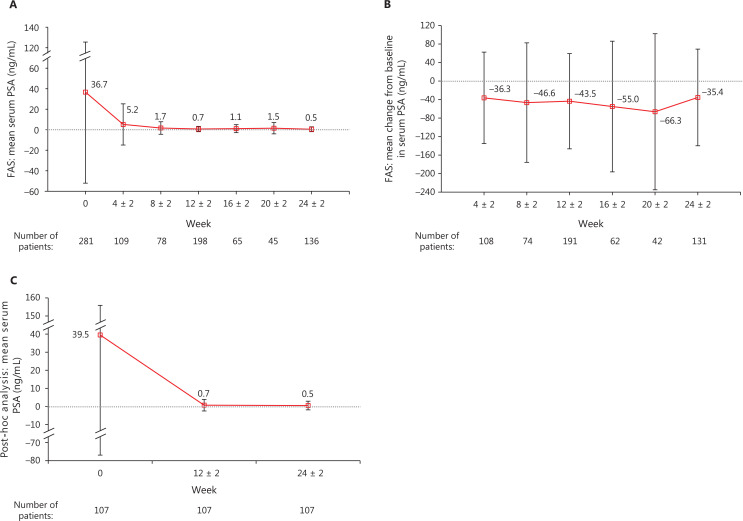
Serum PSA: (A) mean (SD) change from baseline in serum PSA level for the FAS; (B) mean (SD) serum PSA level for the FAS; (C) mean (SD) serum PSA level in the PSA subset. The gray dotted lines in (A), (B) and (C) represent zero. FAS, full analysis set; PSA, prostate-specific antigen; SD, standard deviation.

The mean PSA level (SD) declined from 36.7 ng/mL (88.8 ng/mL) at baseline (*n* = 281) to 5.2 ng/mL (20.1 ng/mL), 0.7 ng/mL (2.6 ng/mL), and 0.5 ng/mL (2.4 ng/mL) at weeks 4 ± 2 (*n* = 109), 12 ± 2 (*n* = 198), and 24 ± 2 (*n* = 136), respectively, in the FAS (**Figure 3A**). The mean change in the serum PSA level from baseline [SD (95% CI)] was −43.5 ng/mL [102.9 ng/mL (−58.2, −28.9 ng/mL)] and −35.4 ng/mL [104.4 ng/mL (−53.5, −17.4 ng/mL)] at weeks 12 ± 2 (*n* = 191) and 24 ± 2 (*n* = 131), respectively (**Figure 3B**).

Subgroups by disease status, RP status, and treatment strategy demonstrated similar trends as the FAS in mean changes from baseline in serum testosterone and PSA levels, mean serum testosterone and PSA levels, and the proportions of patients achieving chemical castration (**Figures S1–S3**).

#### Post-hoc analysis

The mean testosterone level (SD) decreased from 392.2 ng/dL (169.8 ng/dL) in the testosterone subset (*n* = 80) at baseline to 29.6 ng/dL (22.6 ng/dL) and 27.3 ng/dL (23.4 ng/dL) at weeks 12 ± 2 and 24 ± 2 (**Figure 2D**), with a mean change (SD) from baseline of −362.6 ng/dL (173.1 ng/dL) and −365.0 ng/dL (172.9 ng/dL), respectively (**Figure S4**).

The mean PSA level (SD) decreased from 39.5 ng/mL (116.4 ng/mL) at baseline to 0.70 ng/dL (3.2 ng/mL) and 0.5 ng/mL (2.4 ng/mL) in the PSA subset (*n* = 107) at weeks 12 ± 2 and 24 ± 2 (**Figure 3C**), with a mean change (SD) from baseline of −38.8 ng/mL (113.8 ng/mL) and −39.0 ng/mL (114.7 ng/mL), respectively (**Figure S5**).

Between the first and second injections, the proportion of patients who achieved castration in the 10.8-mg group was 86.7% (169/195), which increased to 91.4% (117/128) after the second injection. Between 28 and 84 days after the first and second injections, the rates of castration were 90.7% (97/107) and 87.3% (69/79), respectively.

### Safety

One hundred and seventeen (39.8%) and 109 patients (37.1%) in the FAS reported AEs and treatment-emergent AEs (TEAEs), respectively (**Table 2**). Urinary tract infections (7.1%) and anemia (6.5%) were the most reported TEAEs (**Table 3**). No cardiovascular or sexual-related AEs were reported. Serious AEs (SAEs) occurred in 30 patients (10.2%), all of which were treatment-emergent. One patient receiving goserelin 10.8-mg depot discontinued treatment and died due to an SAE (cerebral hemorrhage), which was assessed to be unrelated to goserelin according to investigator assessment. Adverse drug reactions were reported in 22 patients (7.5%) but none of the adverse drug reactions resulted in treatment discontinuation (**Table 2**).

**Table 2:** Summary of AEs in the FAS

AE, *n* (%)	FAS (*n* = 294)
Any AEs	117 (39.8)
Treatment-emergent AEs (TEAEs)	109 (37.1)
Adverse drug reactions (ADRs)	22 (7.5)
AEs with CTCAE grade ≥ 3	32 (10.9)
TEAEs with CTCAE grade ≥ 3	31 (10.5)
Serious AEs	30 (10.2)
Serious TEAEs	30 (10.2)
Treatment-related serious TEAEs	1 (0.3)
TEAEs leading to death	1 (0.3)
TEAEs leading to treatment discontinuation	1 (0.3)
ADRs leading to treatment discontinuation	0
AEs of interest (cardiovascular, sexual)	0

AE, adverse event; CTCAE, common terminology criteria for adverse events; TEAE, treatment-emergent adverse event.

**Table 3:** Summary of treatment-emergent adverse events (TEAEs) by preferred terms reported in > 1% of patients in the FAS

TEAE, *n* (%)	FAS (*n* = 294)
Urinary tract infection	21 (7.1)
Anemia	19 (6.5)
Pneumonia	8 (2.7)
Hypoproteinemia	8 (2.7)
Diarrhea	6 (2.0)
Hypokalemia	6 (2.0)
Hematuria	6 (2.0)
Hepatic function abnormal	6 (2.0)
Hot flush	6 (2.0)
Constipation	5 (1.7)
Insomnia	5 (1.7)
Upper respiratory tract infection	5 (1.7)
Liver injury	5 (1.7)
Chronic gastritis	4 (1.4)
Hyperlipidemia	4 (1.4)
Blood lactate dehydrogenase increased	4 (1.4)

FAS, full analysis set.

### Follow-up pattern

The mean testing frequency (SD) was 1.6 (1.45) [10.8-mg group, 1.6 (1.41); 3.6-mg group, 2.6 (2.64)] for serum testosterone and 2.2 (1.56) [10.8-mg group, 2.2 (1.53); 3.6-mg group, 3.0 (2.38)] for serum PSA. Seventy-two (24.5%) and 27 patients (9.2%) did not undergo serum testosterone and PSA testing, respectively.

## Discussion

### Summary of results

This is the largest real-world study to date involving the administration of LHRH agonists in Chinese patients with prostate cancer, demonstrating the effectiveness and safety of long-acting goserelin 10.8-mg depot, which was administered to 97.6% of patients in this study. Our study demonstrated that goserelin 10.8-mg depot was effective in suppressing serum testosterone and PSA early during treatment and subsequently maintaining the suppression until the end of treatment, which was consistent across subgroups by disease status, RP status, and treatment strategy. The results also showed that goserelin 10.8-mg depot was well-tolerated in Chinese patients with no new safety issues identified.

### Clinical relevance

A recent survey among urologists in China revealed the urgent need to reduce the injection frequency of LHRH agonists (i.e., adopting use of long-acting LHRH agonists) in patients with prostate cancer^16^. Lower injection frequency could reduce the frequency of hospital visits and medical examinations, thereby reducing healthcare expenditures^16^. Moreover, patients who receive long-acting LHRH agonists have a reduced risk of COVID-19 infection due to less frequent hospital visits. Therefore, the use of long-acting LHRH agonists, such as goserelin 10.8-mg depot, that are effective and safe is highly desirable.

Notably, most patients (61.8%) included in the present study received goserelin 10.8-mg depot as adjuvant therapy after an RP. According to the 2021 Chinese Society of Clinical Oncology (CSCO) guidelines on prostate cancer, adjuvant ADT following an RP is recommended for patients with localized and locally advanced prostate cancer, especially for those patients with persistently elevated serum PSA levels (≥ 0.2 ng/mL) or lymph node metastasis after an RP^7^. Adjuvant ADT after an RP is commonly used in Chinese patients with prostate cancer. A Chinese single-center, retrospective study among patients with prostate cancer who underwent an RP revealed that up to 65.8% (367/558) of patients received adjuvant ADT^22^. Among patients with tumor pathologic stage T_2C_ and ≥ T_3_ after an RP, the proportion of patients receiving adjuvant ADT was 60.2% and 100%, respectively^22^. The present study further demonstrated the effectiveness of adjuvant goserelin 10.8-mg depot after an RP in suppressing serum testosterone and PSA levels in patients with localized or locally advanced prostate cancer (**Figures S1 and S2**).

### Effectiveness – comparison with previous research and interpretation

In this study goserelin demonstrated effectiveness similar to the efficacy observed in clinical trials. In the pooled analysis of two Dutch phase III trials in hormone treatment-naïve patients with advanced prostate cancer, the mean serum testosterone level for patients receiving goserelin 10.8-mg depot decreased from 533.0 ng/dL at baseline to 26.5 and 21.1 ng/dL at weeks 4 and 12, respectively^15,18^. A US trial showed that goserelin 10.8-mg depot treatment reduced the mean serum testosterone level to < 30 ng/dL by week 4 and maintained the suppression until week 26 in patients with advanced prostate cancer^20^. Furthermore, a Japanese trial demonstrated that among hormone treatment-naïve patients with prostate cancer, goserelin 10.8-mg depot treatment decreased the mean serum testosterone from 507 ng/dL at baseline to 10 ng/dL at week 24, which was slightly lower than the level reported in this study (29.2 ng/dL). The mean serum PSA decreased from 52.4 ng/mL at baseline to 0.9 ng/mL at week 24, which was higher than the level reported in this study (0.5 ng/mL)^19^. Factors that contributed to these differences are complex. Notably, the baseline patient characteristics differed substantially between the two studies. Specifically, patients included in the Japanese trial were older (mean age at baseline, 75.0 *vs.* 70.2 years) and had a higher proportion with clinical stage T1–2 (64.8% *vs.* 36.7%)^19^. Moreover, the Japanese trial included patients with metastatic disease (clinical stage M1, 24.1%)^19^; patients with metastatic disease were excluded from the current study. The influence of these factors on testosterone and PSA levels during ADT requires further research. Nevertheless, the decreasing trends in both serum testosterone and PSA levels were similar between the two studies. Additionally, the results reported in this study were also consistent with other real-world studies on goserelin 10.8-mg depot and clinical trials on goserelin 3.6-mg depot^21,23,24^.

The effectiveness of goserelin in this study was also largely comparable to the observed efficacy of other LHRH agonists. In an international phase III trial among patients with advanced prostate cancer, the sustained serum testosterone level < 50 ng/dL from day 29 through week 48 was 88.8% in patients receiving leuprolide treatment every 3 months^25^, a proportion similar to the castration rate (87.7%–95.7%; **Figure 2C**) from week 8 ± 2 in the current study. In a Korean retrospective study among patients with advanced prostate cancer, the castration rates (serum testosterone level < 50 ng/dL) at month 6 were 100% in all treatment groups [goserelin 11.34 mg, leuprolide 11.25 mg, and triptorelin 11.25 mg (all administered every 3 months)]^26^, which was slightly higher than the 90.2% castration rate at week 24 ± 2 in the current study.

The proportion of patients with testosterone escape (serum testosterone ≥ 50 ng/dL) in this study was approximately 10% throughout the study period, except at week 4 ± 2 (16.2%). The relatively higher rate of testosterone escape at week 4 ± 2 could be because 59.5% (44/74) of patients who received testosterone tests within this time window were tested < 21 days after treatment initiation, which is earlier than the timepoint when testosterone suppression could be achieved by goserelin according to previous trials^17^. Testosterone escape is not uncommon during LHRH agonist treatment in clinical practice, with an estimated incidence of 6.9% based on a meta-analysis^27^. A Canadian retrospective study reported that the rate of testosterone escape per patient during the course of treatment with goserelin was 10.5%, which was similar to intramuscular leuprolide (11.5%) and triptorelin (6.7%)^28^, and comparable to the results of this study. The testosterone escape observed in this study could be attributed to the tapering-off of drug level towards the end of each administration cycle^27^, which also might partially explain the slight decrease in the proportion of patients achieving chemical castration from week 8 ± 2 (91.5%) to week 12 ± 2 (87.7%), and from week 16 ± 2 (92.9%) to week 24 ± 2 (90.2%). Another basis for testosterone escape could be the varying accuracy of testosterone measurements between studies. Administration or device failures and the development of ADT resistance could further contribute to testosterone escape^27^.

### Safety – comparison with previous research and interpretation

Goserelin demonstrated good tolerability in Chinese patients with prostate cancer in this study. The overall incidence of TEAEs was similar to the incidence reported in an Italian real-world study (37.1% *vs.* 37.3%)^23^ but lower than the reported incidence in several phase III trials^15,17,18,20^, which was likely because AEs are less closely monitored in real-world settings than in clinical trials. Urinary tract infections and anemia were the most common TEAEs in this study. The high incidence (7.1%) of urinary tract infections could be attributed to the high proportion [38.1% (112/294)] of patients who underwent an RP, after which a urinary tract infection is a common complication^29,30^. The 6.5% incidence of anemia was not unexpected because hemoglobin decreased was reported in > 25% of patients receiving goserelin treatment in the aforementioned Japanese trial^19^. However, hot flush, reported to be the most common TEAE in previous trials (incidence, 51%–70%)^17,18,20,23^, was uncommon (2.0%) in this study, possibly because patients were less likely to disclose mild AEs in real-world settings and not questioned specifically for the symptom as in clinical trials. Moreover, the incidence of ADRs in this study was also similar to the aforementioned Italian real-world study (7.5% *vs.* 6.8%)^23^.

### Monitoring patterns

It is necessary to monitor serum testosterone and PSA levels during ADT so that timely intervention can be implemented upon treatment failure. The 2021 CSCO guidelines on prostate cancer recommend determining testosterone and PSA levels every 3–6 months^7^. Similar recommendations have been made in prostate cancer guidelines from the National Comprehensive Cancer Network (NCCN) and the European Association of Urology (EAU)^8,31^. The testing frequencies reported in this study were consistent with the recommendations by the abovementioned guidelines. However, a considerable proportion (24.1%) of patients failed to undergo any follow-up testing for serum testosterone, indicating that monitoring of these patients needs to be improved.

### Limitations

The study had some limitations. First, the long-term efficacy, safety, and survival outcomes associated with goserelin treatment of prostate cancer could not be assessed due to the short study duration. Hence, further studies with a longer duration of follow-up are warranted to confirm the survival benefits of goserelin 10.8-mg depot in Chinese patients with prostate cancer. A second limitation was that the lack of targeted monitoring in real-world settings might have resulted in underreporting of AEs.

Another limitation was that the center effect was not accounted for in our study. Thus, due to variability between centers in patient characteristics, clinical practice, and provider expertise, uncertainty in study results might arise. However, our study was non-interventional and was not designed to formally compare the clinical outcomes associated with different treatments, between patients enrolled from different centers, or between patient groups with different baseline characteristics. Therefore, the center effect was not considered a major concern for this study. Moreover, due to the large number of centers (*n* = 29) involved, the number of patients recruited from some centers was small. There were also practical difficulties in comprehensively identifying and adjusting for potential confounders, such as differences in healthcare infrastructure, provider experience, and regional factors, many of which were not readily quantifiable or available. Therefore, detecting a true center effect was not feasible.

Additionally, the study was limited by the lack of formal comparative analysis between patient subgroups. However, the results of the study indicated that the mean testosterone and PSA levels of patients in different subgroups, as well as the respective standard deviations, were highly similar during the follow-up period (**Figure S2**), which provided sufficient evidence to conclude that treatment efficacy was similar across subgroups. Moreover, it was not feasible to perform a meaningful comparative analysis based on goserelin regimen due to the small number of patients who received the 3.6-mg formulation (*n* = 7). Thus, the lack of a formal comparative analysis in this study did not constitute a major concern.

## Conclusions

In conclusion, goserelin 10.8-mg depot demonstrated good effectiveness and tolerability in Chinese patients with localized or locally advanced prostate cancer, with > 90% of patients achieving chemical castration by the end of the study. The real-world testing frequencies of serum testosterone and PSA were consistent with the current guideline recommendations.

## Data Availability

The data generated in this study are available upon reasonable request from the corresponding author.
