# Supplementary material for: Real-world effectiveness and safety of goserelin 10.8-mg depot in Chinese patients with localized or locally advanced prostate cancer
Source: Cancer Biol Med. 2024 Feb 5;20(12):1047–59. doi: 10.20892/j.issn.2095-3941.2023.0335 (PMC10845927; doi:10.20892/j.issn.2095-3941.2023.0335)
Supplement: Supplementary file 1 [file cbm-20-1047-s001.pdf]

Supplementary materials

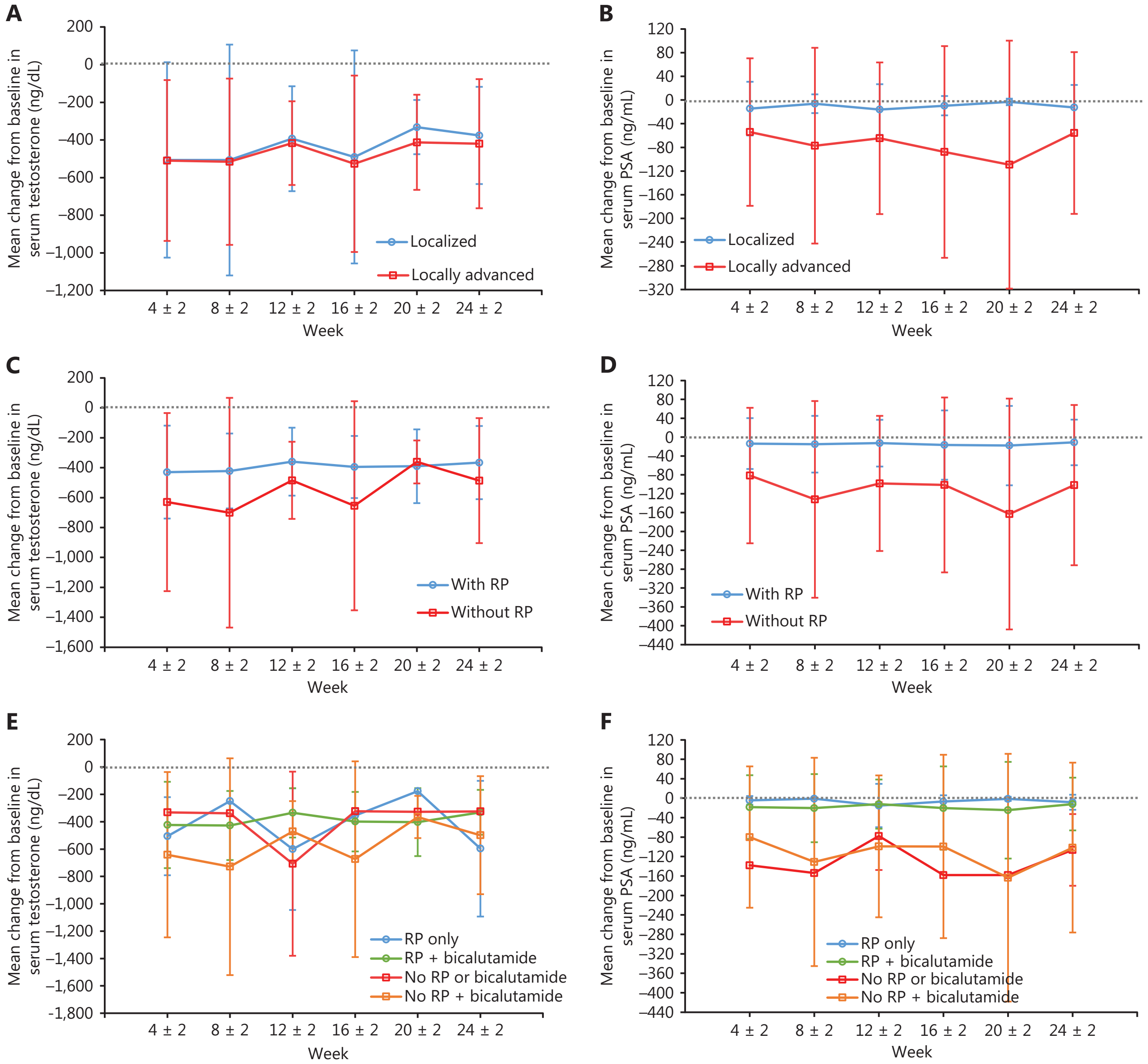

Figure S1 Mean (SD) change from baseline in serum testosterone and PSA levels for subgroups by disease status (A, B), RP status (C, D), and treatment strategy (E, F). The gray dotted lines represent zero in all figures. FAS, full analysis set; SD, standard deviation. PSA, prostate specific antigen; RP, radical prostatectomy.

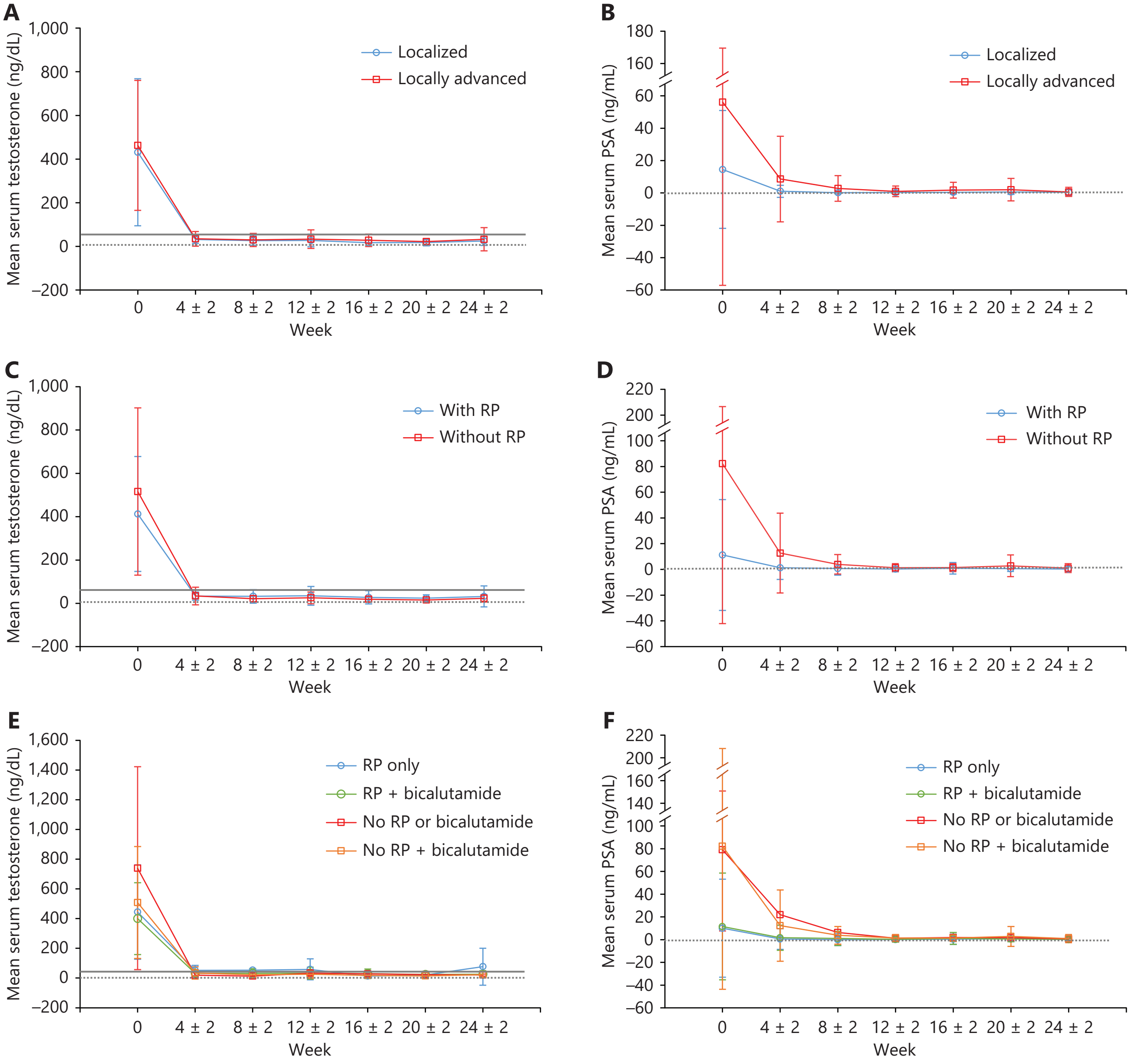

Figure S2 Mean (SD) serum testosterone and PSA levels for subgroups by disease status (A, B), RP status (C, D), and treatment strategy (E, F). The gray dotted lines represent zero in all figures, and the gray solid lines in (A), (C) and (E) represent the serum testosterone level of 50 ng/dL. FAS, full analysis set; SD, standard deviation. PSA, prostate-specific antigen; RP, radical prostatectomy.

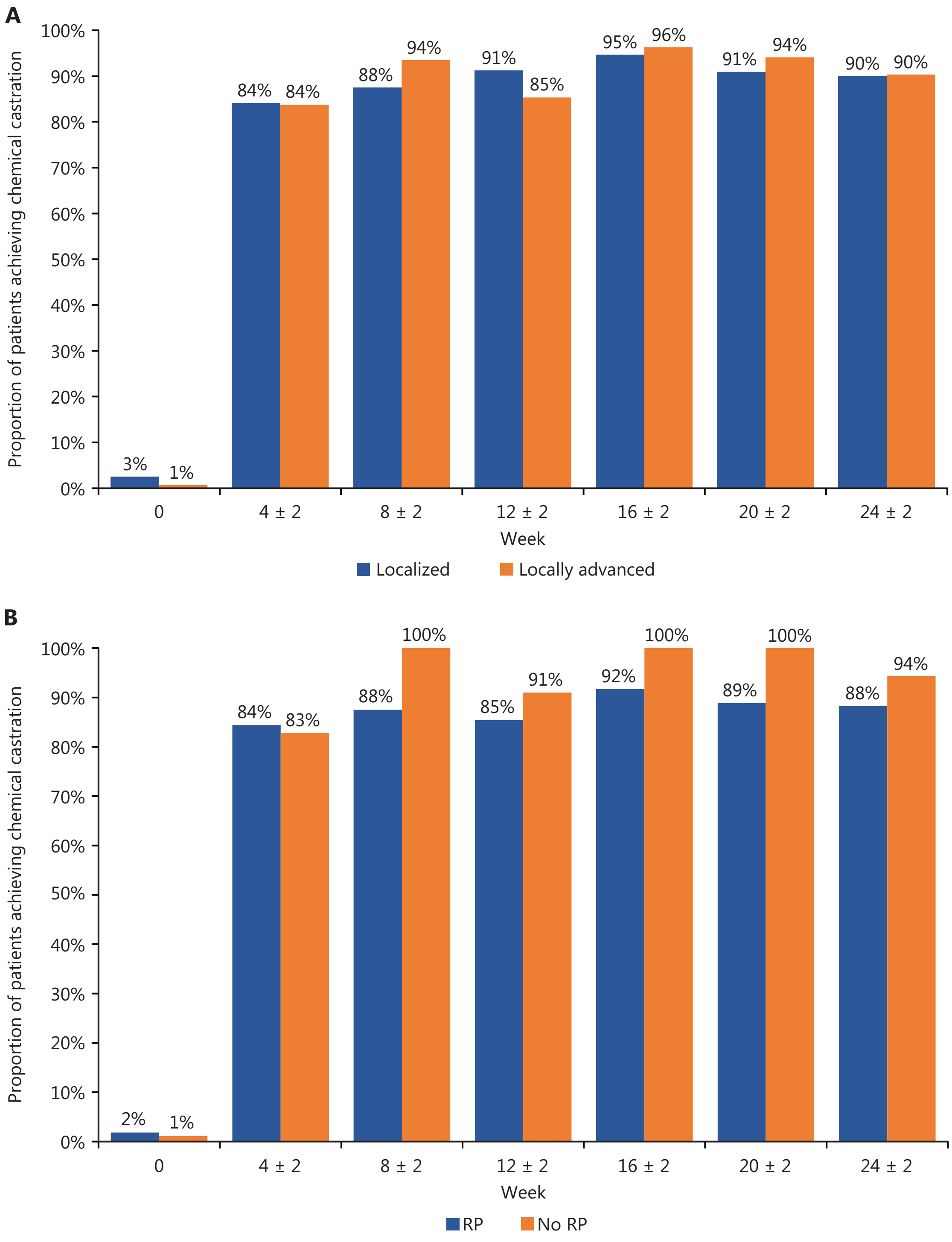

Figure S3 Proportions of patients achieving chemical castration (serum testosterone < 50 ng/dL) for subgroups by (A) disease status and (B) RP status. RP, radical prostatectomy.

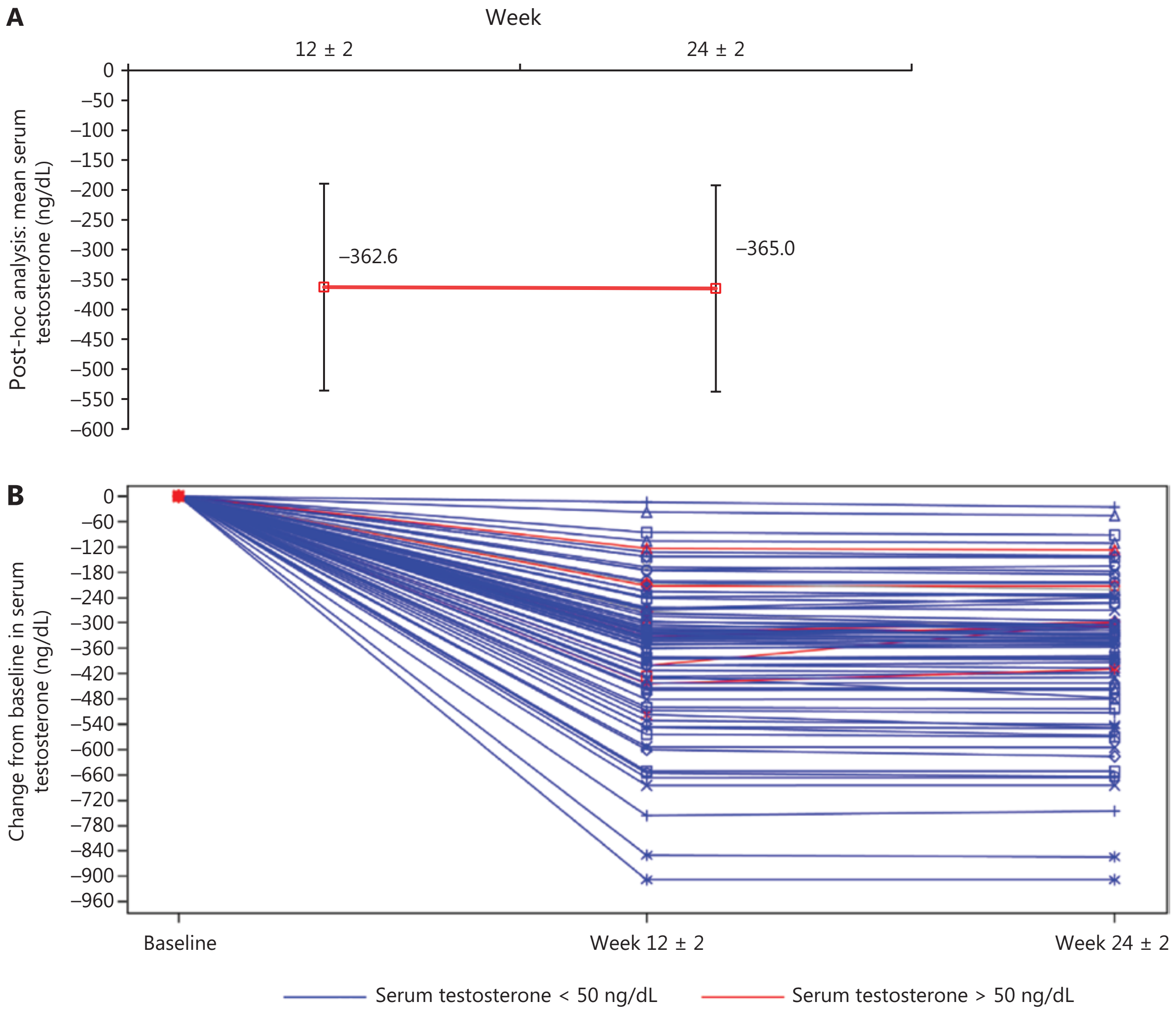

Figure S4 Serum testosterone in the testosterone subset: (A) mean (SD) change from baseline in serum testosterone level and (B) spider plot for the serum testosterone levels of individual patients.

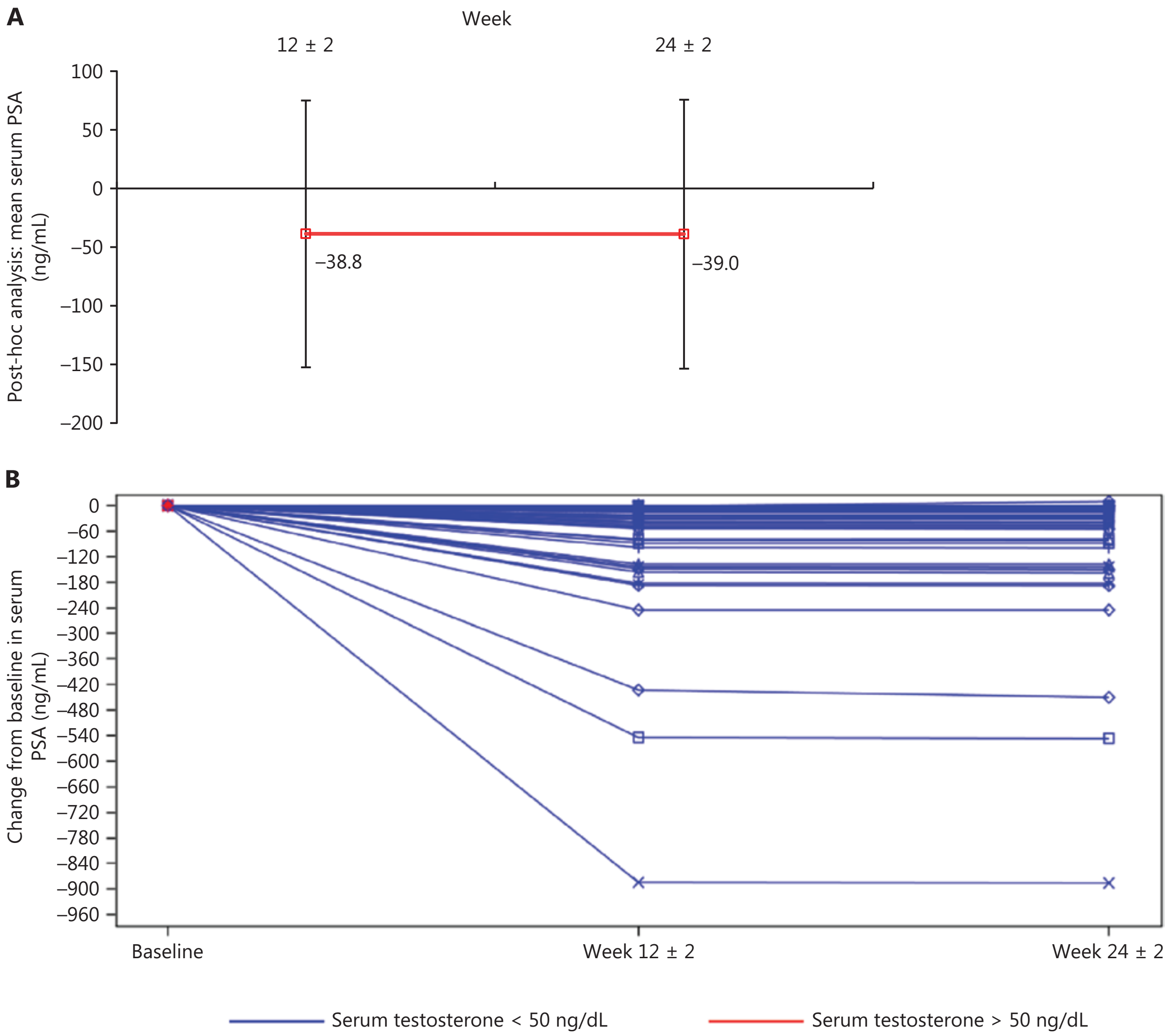

Figure S5 Serum PSA in the PSA subset: (A) mean (SD) change from baseline in serum PSA level and (B) spider plot for the serum PSA levels of individual patients. The gray dotted line in (A) represents zero. FAS, full analysis set; SD, standard deviation. PSA, prostate-specific antigen.
